# A genome-wide association study uncovers a critical role of the *RsPAP2* gene in red-skinned *Raphanus sativus* L.

**DOI:** 10.1038/s41438-020-00385-y

**Published:** 2020-09-24

**Authors:** Lianxue Fan, Yan Wang, Liang Xu, Mingjia Tang, Xiaoli Zhang, Jiali Ying, Cui Li, Junhui Dong, Liwang Liu

**Affiliations:** National Key Laboratory of Crop Genetics and Germplasm Enhancement, Key Laboratory of Horticultural Crop Biology and Genetic Improvement (East China) of MOAR, College of Horticulture, Nanjing Agricultural University, 210095 Nanjing, PR China

**Keywords:** Genome-wide association studies, Secondary metabolism, Transcription, Gene regulation

## Abstract

Radish (*Raphanus sativus* L.) taproot contains high concentrations of flavonoids, including anthocyanins (ATCs), in red-skinned genotypes. However, little information on the genetic regulation of ATC biosynthesis in radish is available. A genome-wide association study of radish red skin color was conducted using whole-genome sequencing data derived from 179 radish genotypes. The R2R3-MYB transcription factor *production of anthocyanin pigment 2* (*PAP2*) gene was found in the region associated with a leading SNP located on chromosome 2. The amino acid sequence encoded by the *RsPAP2* gene was different from those of the other published *RsMYB* genes responsible for the red skin color of radish. The overexpression of the *RsPAP2* gene resulted in ATC accumulation in *Arabidopsis* and radish, which was accompanied by the upregulation of several ATC-related structural genes. RsPAP2 was found to bind the *RsUFGT* and *RsTT8* promoters, as shown by a dual-luciferase reporter system and a yeast one-hybrid assay. The promoter activities of the *RsANS*, *RsCHI*, *RsPAL*, and *RsUFGT* genes could be strongly activated by coinfiltration with *RsPAP2* and *RsTT8*. These findings showed the effectiveness of GWAS in identifying candidate genes in radish and demonstrated that RsPAP2 could (either directly or together with its cofactor RsTT8) regulate the transcript levels of ATC-related genes to promote ATC biosynthesis, facilitating the genetic enhancement of ATC contents and other related traits in radish.

## Introduction

Radish (*Raphanus sativus* L.) taproot is a valuable source of phytochemicals such as flavonoids. Anthocyanins (ATCs) are a subgroup of flavonoids that exhibit strong antioxidant activity that is beneficial for human health^1^. Previous studies have reported that the major components of ATC pigments in red radish epidermal tissue are acylated pelargonidin glycosides with a combination of p-coumaric, ferulic, or malonic acids^2,3^. Pelargonidin exhibits an orange-red hue, and acylation is expected to shift the hue to red and improve stability^2^, leading to the formation of red skin in radish.

ATCs are synthesized via the flavonoid branch of the general phenylpropanoid pathway^4^. The initial steps of the phenylpropanoid pathway involve phenylalanine ammonia lyase (PAL), cinnamate 4-hydroxylase (C4H), and 4-coumarate-CoA ligase (4CL). Subsequently, the flavonoid pathway is initiated by the condensation of one molecule of 4-coumaroyl-CoA with three molecules of malonyl-CoA by chalcone synthase (CHS). The successive steps catalyzed by chalcone isomerase (CHI), flavanone 3-hydroxylase (F3H), dihydroflavonol-4-reductase (DFR), anthocyanidin synthase (ANS), and flavonoid 3-oglucosyltransferase (UFGT) lead to the production of ATC pigments^5,6^. These crucial structural genes in the ATC biosynthesis pathway have been isolated and well characterized in plant species such as *Arabidopsis*, apple, eggplant, and grape^1,7–10^.

ATC biosynthesis is known to be transcriptionally regulated by R2R3-MYB transcription factors (TFs), basic helix-loop-helix (bHLH) TFs and WD40 repeat proteins, which normally form MYB–bHLH–WD40 complexes^1,8^. The conserved complex interacts with a 7-bp MYB-recognizing element (MRE, “ANCNNCC”) and a 6-bp bHLH-recognizing element (BRE, “CACN[A/C/T][G/T]”) in the promoter regions of ATC structural genes in various plant species^11^. Among these regulators, MYB TFs are considered essential regulators of ATC production; these TFs include MYB75 (production of anthocyanin pigment 1, PAP1), MYB90 (PAP2), MYB113 and MYB114 in *Arabidopsis*^12–14^, MdMYB10, MdMYBPA1 and MdMYB308L in apple^9,15,16^, and PyMYB10 and PyMYB114 in Chinese pear^17,18^. In addition, a few members of the bHLH TF family, such as AtTT8 (bHLH42) in *Arabidopsis* and StbHLH1 in potato, also result in enhanced activity at promoters containing a *cis*-regulatory element that is conserved in several ATC biosynthetic genes^19,20^. Most of the structural genes known to be involved in ATC biosynthesis have been reported in radish^21^; however, only a few TFs have been clearly identified as being related to the transcriptional regulation of the ATC biosynthesis pathway in radish, such as RsMYB1, RsMYB1a, RsMYB90, and RsTT8^22–27^.

Genome-wide association studies (GWASs) are a valuable, powerful tool for dissecting the genetic basis of complex traits in plants^28,29^. The GWA mapping of 360 natural genotypes of *Arabidopsis* revealed that *MYB90* was the major gene responsible for the wide variation in ATC accumulation^14^. A total of six candidate loci responsible for ATC variation in lettuce leaves were identified using a GWAS approach^29^. The peach *F3H* gene was found in the associated region of a GWAS signal for flesh color^30^. Moreover, GWAS has been applied to investigate ATC pigmentation and fruit color in eggplant^31^, grape^32^, and rose^33^. These studies indicate the usefulness of GWAS for deciphering the genetic control of ATC biosynthesis and red skin color in radish taproots.

In this study, to identify key ATC-specific structural genes or regulatory factors and better understand the genetic regulation of red skin color in radish, a GWAS for radish red skin color was conducted in a diverse population of 179 radish genotypes. An R2R3-MYB TF, *RsPAP2*, was identified as being associated with ATC biosynthesis in red-skinned radish taproot using high-quality single nucleotide polymorphisms (SNPs). The overexpression of the *RsPAP2* gene in *Arabidopsis* plants and radish cotyledons positively promoted red pigmentation. RsPAP2 could bind the promoters of several ATC biosynthetic genes, either directly or with its partner RsTT8, to promote ATC biosynthesis, indicating that the *RsPAP2* gene is a critical transcriptional activator for ATC production in radish. This study provides valuable information for the clarification of the molecular mechanism underlying taproot skin coloration and will facilitate the genetic improvement of ATC-associated traits in radish breeding programs.

## Results

### SNP genotyping, population structure, and linkage disequilibrium (LD) analyses

A total of 179 cultivated radish genotypes were selected for whole-genome resequencing analysis (Table S1), generating 819.71 Gb of clean reads (~4.58 Gb for each sample on average). The clean reads from each genotype were mapped to the “WK10039” radish genome^34^. The mapping rate varied from 70.48 to 94.73% among different genotypes, with an average of 89.70% (Table S1). A total of 1,222,458 high-quality SNPs were detected, among which 588,896 SNPs (48.17%) were located within genes (Fig. S1a). The SNP density was consistent with the density of genes on the nine radish chromosomes (Fig. S2). Moreover, a total of 98,730 SNPs were suggested to have a potential impact on gene function as splicing, stop-gain, stop-loss, and nonsynonymous variants (Fig. S1b).

**Fig. 1:**
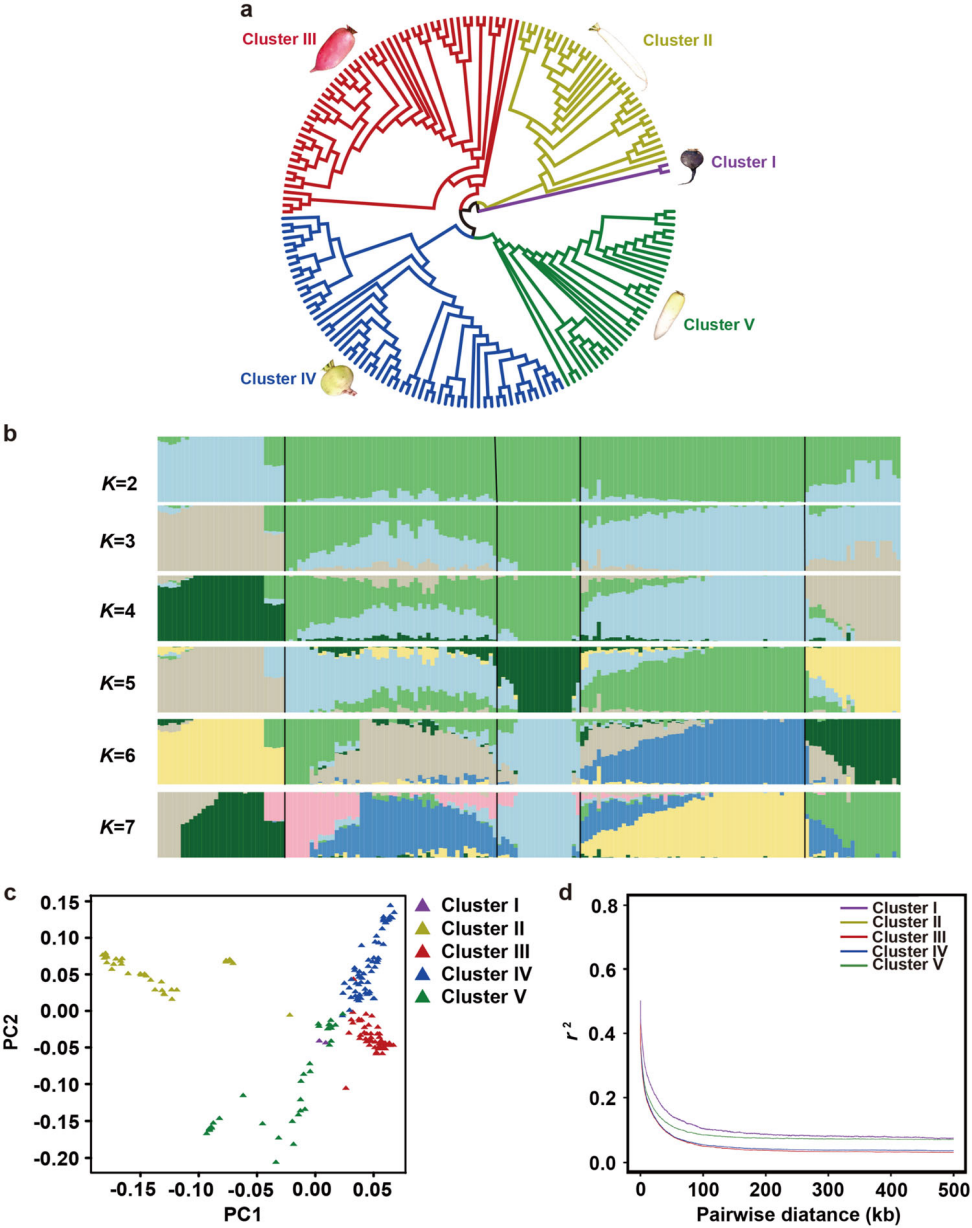
Population structure of radish genotypes. **a** Neighbor-joining phylogenetic tree. **b** Population structure analysis with different numbers of clusters (*K* = 2–7). **c** PCA of all radish genotypes. The first two principal components (PC1 and PC2) were used to visualize the relationships among individuals and groups. Each point represents an independent radish genotype. **d** Decay of LD in five clusters measured by *r*^2^

Based on the SNP data, a neighbor-joining tree was constructed to show the phylogenetic relationships among 179 cultivated radish genotypes (Fig. 1a). The “NAU-145” and “NAU-175” genotypes, with black skin, were clustered into one clade (Cluster I), while the remaining 177 genotypes from Asia were divided into four other clusters. Cluster II contained 31 long-white genotypes and the red-skinned genotype “NAU-017”. Fifty red-skinned genotypes and three landraces (“NAU-041”, “NAU-083”, and “NAU-155”) were classified into Cluster III. Cluster IV included 59 landraces from China, while Cluster V consisted of 5 genotypes from Japan (“NAU-116”, “NAU-124”, “NAU-133”, “NAU-136”, and “NAU-140”) and 28 other accessions. A structure analysis showed the minimum cross-validation error at *K* = 5, which was determined to be the optimum *K*-value for the radish population (Fig. 1b). Principal component analysis (PCA) was performed, and PC1 and PC2 differentiated five radish clusters (Fig. 1c), which was supported by both phylogenetic and structural analyses. In addition, the PCA plot indicated that the long-white genotype from Cluster II was more distantly related to the other genotypes, especially the red-skinned genotype from Cluster III, consistent with their phenotypic variations in skin color.

The LD level was measured as the physical distance at which *r*^2^ decreased to 0.20, and LD decay was estimated to be 7 kb when all genotypes were analyzed. LD decay in the five clusters varied from 2.5 to 22.8 kb, with the largest (22.8 kb) decay observed for the genotypes with black skin (Fig. 1d).

### GWAS analysis for red skin color in radish taproots

**Fig. 2:**
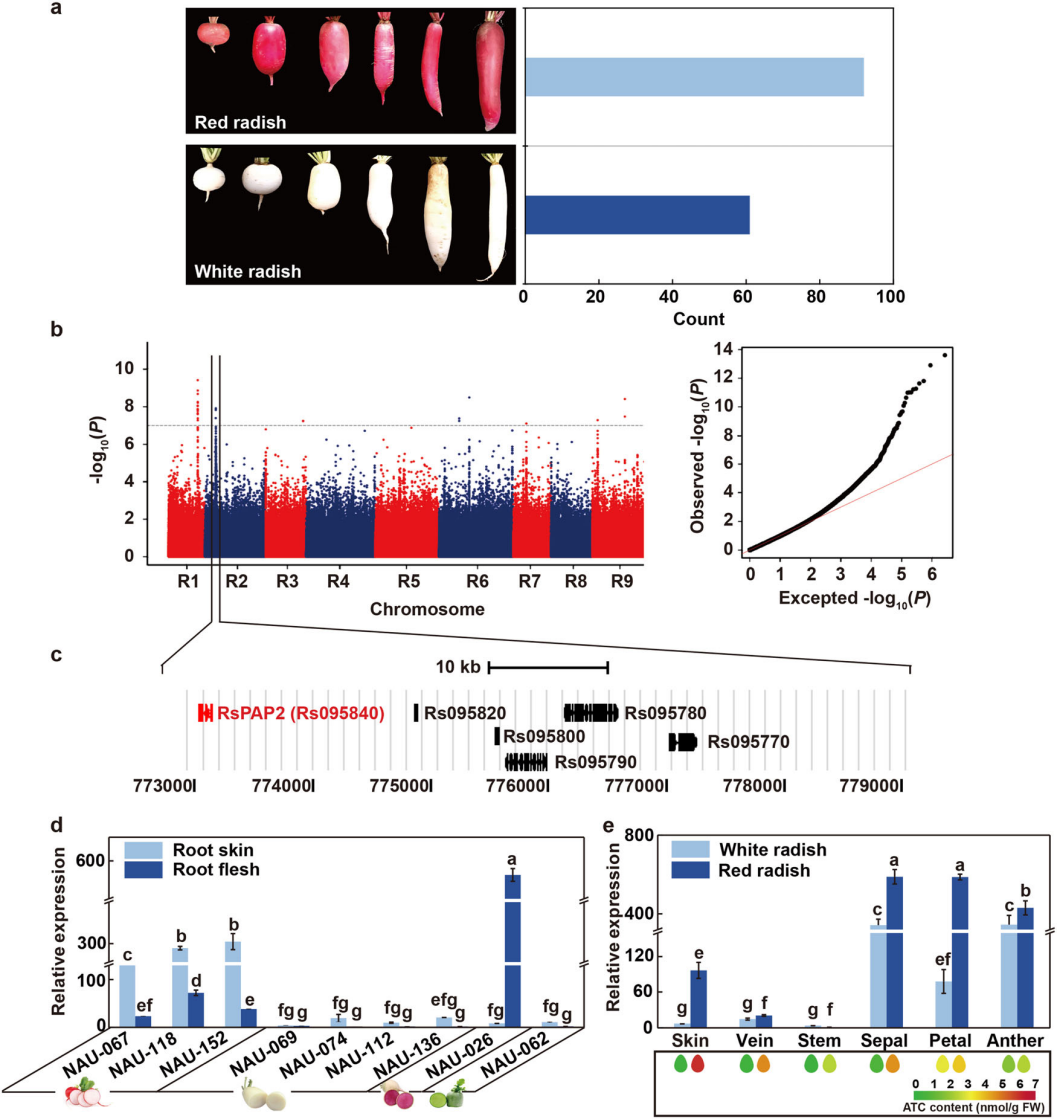
GWAS analysis for red skin color in radish. **a** Frequency distribution of skin color (red and white) variations across 179 radish genotypes. **b** Manhattan plots (left) and quantile-quantile plots (right) depicting results of the GWAS for red skin color. The *x*-axis depicts the physical location of SNPs across the 9 chromosomes of radish, and the *y-*axis depicts the −log_10_ (*P* value). **c** All genes positioned in the region around the leading SNPs on Chr2. **d**, **e** Relative expression patterns of the candidate gene *RsPAP2* in the roots of nine genotypes and in different tissues of the white- and red-skinned genotypes (“NAU-069” and “NAU-067”), respectively. Heatmap showing the total ATC content of different tissues from “NAU-069” and “NAU-067”, respectively. Error bars indicate the standard deviation (SD) from at least three replicates. Letters represent significant differences at the 0.05 level based on Duncan’s test

Considering the phylogenetic relationships and the variations in skin color among the 179 radish genotypes, 65 white- and 95 red-skinned genotypes were singled out for the association analysis of red skin color (Fig. 2a). A total of 14 significantly associated SNP signals containing 26 SNPs were detected at the applied significance threshold (−log_10_ *P* > 7). Notably, two leading SNPs were observed on chromosomes (Chrs) 1 and 2 (Fig. 2b and Table S2), which were located in an unknown gene (Rs033130) and a calmodulin-binding transcription activator (Rs095780), respectively. However, both of these SNPs were synonymous variation.

Based on the LD level, a total of 39 candidate genes were identified in the space defined by the ±10 kb regions on either side of the 14 peak SNPs, such as the *production of anthocyanin pigment 2* (*PAP2*, Rs095840), *Myb domain protein 92* (Rs144280), and *zinc finger protein-related* (Rs144260) genes (Table S2). Remarkably, *RsPAP2*, located on Chr2, was associated with one of the top two SNPs (Fig. 2c), indicating that it might be a potential candidate responsible for the red skin color of radish.

### Identification of the candidate gene *RsPAP2* responsible for red skin color

The *PAP2* gene, also known as the “*MYB90* TF”, is responsible for determining ATC contents in *Arabidopsis* and rose petals^14,33^. A MYB90 TF, *RsPAP2*, was also detected in the associated region of the leading SNP located on Chr2 (Fig. 2b, c and Table S2). The 720-bp coding sequence of the *RsPAP2* gene was isolated from the red-skinned radish genotype “NAU-067”, encoding a polypeptide of 239 amino acids. In the phylogenetic analysis of ATC-related R2R3-MYB proteins from radish and *Arabidopsis*, RsPAP2 from “NAU-067” was clustered into a clade together with the published radish MYBs and AtMYB75/PAP1, AtMYB90/PAP2, and AtMYB113 from *Arabidopsis* (Fig. S3a). The published radish MYBs were further divided into four clades (RsMYB I, RsMYB II, RsMYB III, and RsMYB IV), but RsPAP2 did not belong to any of these clades (Fig. S3a), indicating that they were different genes. In particular, RsPAP2 and RsMYB1.1 (CX16Q-25-2), located on Chr2, showed significant differences between their amino acid sequences (Fig. S4). Moreover, the amino acid sequence of RsMYB1.1 was subjected to BLAST searches against the radish genome of WK10039, and it was found that RsMYB1.1 was located at the position of Rs094840 (Chr2: 8,425,153–8,426,787 bp), which is different from that of the RsPAP2 gene (Rs095840, Chr2: 7,730,000–7,731,000 bp) (Fig. S3b). In addition, the RsPAP2 protein contained the complete R2R3 domain at the amino terminus. A bHLH motif ([D/E]Lx_2_[R/K]x_3_Lx_6_Lx_3_R) was found in the R3 domain (Fig. S4), suggesting that RsPAP2 belonged to an R2R3-MYB family member. In addition, a total of three nonsynonymous SNPs were identified in the coding region of *RsPAP2* among five red-skinned and five white-skinned radish genotypes, which resulted in three amino acid substitutions in the protein sequence of RsPAP2 (Fig. S5). However, two substitutions were only identified in “NAU-009”, “NAU-090”, and “NAU-103”, while the other substitution was not located in a conserved domain (Fig. S5). Therefore, these substitutions are unlikely to affect protein activity.

### The expression pattern of *RsPAP2* is consistent with ATC content in radish

The transcript abundance of the *RsPAP2* gene in nine radish genotypes with different skin and flesh colors was measured by real-time quantitative polymerase chain reaction (RT-qPCR) analysis (Fig. 2d). The results showed that the transcriptional levels of *RsPAP2* in root skin were 6.5-, 4.0-, and 8.0-fold higher than those in root flesh among the three red-skinned genotypes “NAU-067”, “NAU-118”, and “NAU-152”, respectively (Fig. 2d). In the genotype “NAU-026” with green skin and red flesh, *RsPAP2* was significantly upregulated in root flesh compared with root skin (Fig. 2d). However, the expression levels of *RsPAP2* in four genotypes with white skin and flesh (“NAU-069”, “NAU-074”, “NAU-112”, and “NAU-136”) and one radish with green skin and flesh (“NAU-062”) exhibited no significant difference in the skin or flesh (Fig. 2d). Furthermore, the *RsPAP2* gene exhibited high expression levels in tissues with abundant ATC accumulation, such as the root skin, leaf veins, stem, sepals, petals, and anthers of the red-skinned genotypes (Fig. 2e). These results indicated that the *RsPAP2* gene is an important regulator of the red skin color of radish taproots.

### Stable expression of *RsPAP2* promotes ATC biosynthesis in *Arabidopsis*

**Fig. 3:**
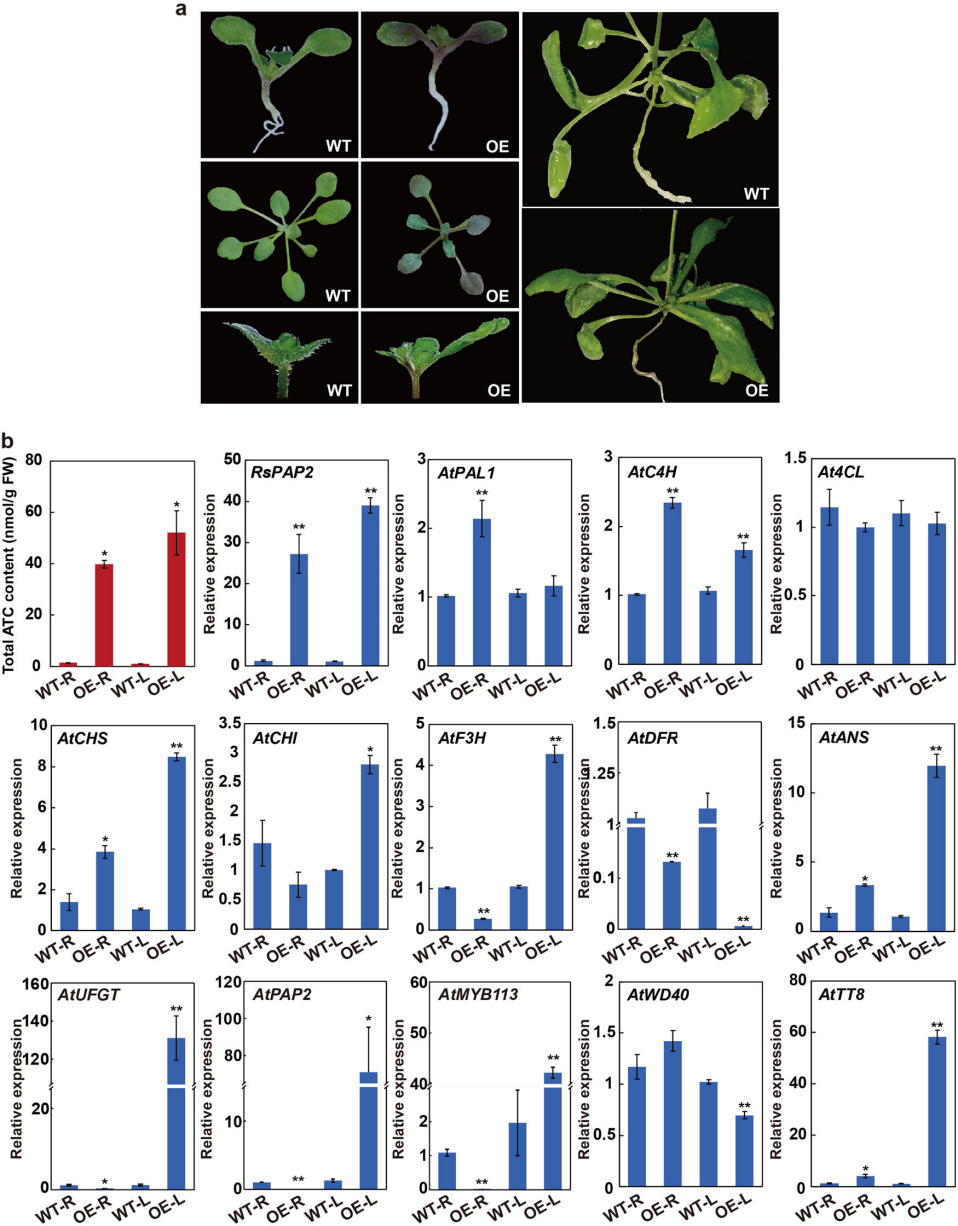
Functional analysis of *RsPAP2* in *Arabidopsis*. **a** Phenotype of *RsPAP2*-overexpressing *Arabidopsis* plants. The 15-day-old leaves, 30-day-old leaves, roots, and the stems near the inflorescences of the transgenic plants were purple. **b** Total ATC contents (top-left) and relative expression levels of *RsPAP2* and 13 endogenous ATC-related genes in the roots (R) and leaves (L) of 30-day-old transgenic *Arabidopsis*. WT wild-type *Arabidopsis* plants, OE *RsPAP2*-overexpressing *Arabidopsis* plants. The error bar represents the SD from three biological replicates in three parallel experiments, and statistical significance was determined using Student’s *t* test. *p < 0.05; **p < 0.01

To verify its potential function in ATC biosynthesis, an overexpression vector containing *RsPAP2* driven by the CaMV35S promoter was transformed into wild-type *Arabidopsis* plants (WTs) (Fig. S6). Among the 19 transgenic *RsPAP2*-overexpressing *Arabidopsis* lines (*RsPAP2*-OEs), eight displayed different levels of purple coloration on the leaves, roots, and stems near inflorescences, but no purple or red coloration was observed in other organs (Fig. 3a). Consistent with the expression levels of the *RsPAP2* transgene, the total ATC contents in the roots and leaves of *RsPAP2*-OEs were ~25.9- and 47.8-fold higher than those in the WTs, respectively (Fig. 3b). These results indicated that the heterologous expression of *RsPAP2* promoted ATC accumulation in the roots and leaves of *Arabidopsis*.

To further investigate the molecular basis of the reddish phenotypes of the different tissues in the transgenic lines, 13 key endogenous genes, including nine structural and four regulatory genes related to ATC biosynthesis, were analyzed by RT-qPCR in the roots and leaves of *RsPAP2*-OE plants (Fig. 3b). The results showed that *AtC4H*, *AtCHS*, *AtANS*, and *AtTT8* were significantly upregulated in both the roots and leaves of the *RsPAP2*-OEs compared with the WTs, whereas *AtDFR* was downregulated. *AtPAL1* and *AtCHI* were specifically upregulated in the roots and leaves, respectively. Interestingly, four other ATC-related genes, *AtF3H*, *AtUFGT*, *AtPAP2*, and *AtMYB113*, exhibited upregulated expression in the leaves of the *RsPAP2*-OE plants but showed the opposite expression pattern in the roots. These findings indicated that RsPAP2 could activate the ATC biosynthesis pathway at the transcriptional level in *Arabidopsis* plants.

### RsPAP2 induces ATC biosynthesis in radish cotyledons by promoting the expression of ATC-related genes

**Fig. 4:**
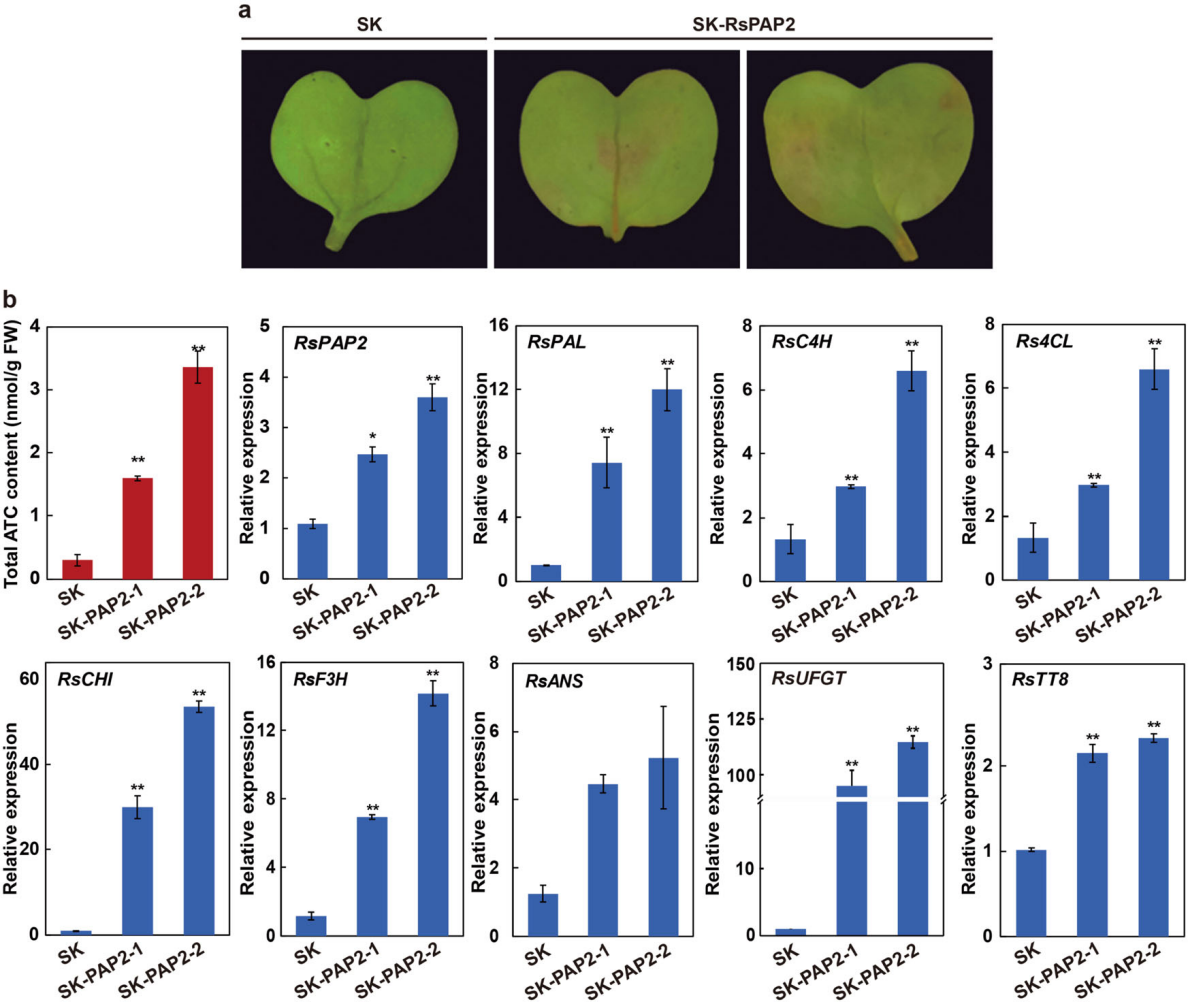
Transient transformation analysis of radish cotyledons. **a** Radish cotyledon transiently expressing the empty vector (SK) and *RsPAP2* (SK-RsPAP2). **b** Total ATC contents (top-left) and relative expression levels of *RsPAP2* and eight ATC-related genes in reddish cotyledons. Error bars represent the SD from three biological replicates in three parallel experiments assessed by RT-qPCR. Statistical significance was determined using Student’s *t* test. *p < 0.05; **p < 0.01

The transient transformation of radish with *RsPAP2* was further performed to investigate its role in ATC production. Some pigmentation was observed after transformation with *RsPAP2* at the infiltration sites in radish cotyledons (Fig. 4a). Consistent with the visual phenotype, the total ATC contents of the reddish cotyledons were 4.3- and 10.1-fold higher than those in the control transformed with the empty vector. As expected, the expression levels of the *RsPAP2* gene in the infiltrated cotyledons were 1.3 and 2.3 times higher than those in the control (Fig. 4b). To determine how the radish cotyledons produce red pigments, eight ACT-related genes were selected for RT-qPCR analysis (Fig. 4b). Six structural genes (*RsPAL*, *RsC4H*, *Rs4CL*, *RsCHI*, *RsF3H*, and *RsUFGT*) were significantly upregulated in the reddish cotyledons compared with the control. In addition, the expression of *RsTT8*, a regulator of ATC biosynthesis, was upregulated in cotyledons overexpressing *RsPAP2* (Fig. 4b), demonstrating that *RsPAP2* induces ATC production via the transcriptional activation of ATC biosynthetic genes in radish.

**Fig. 5:**
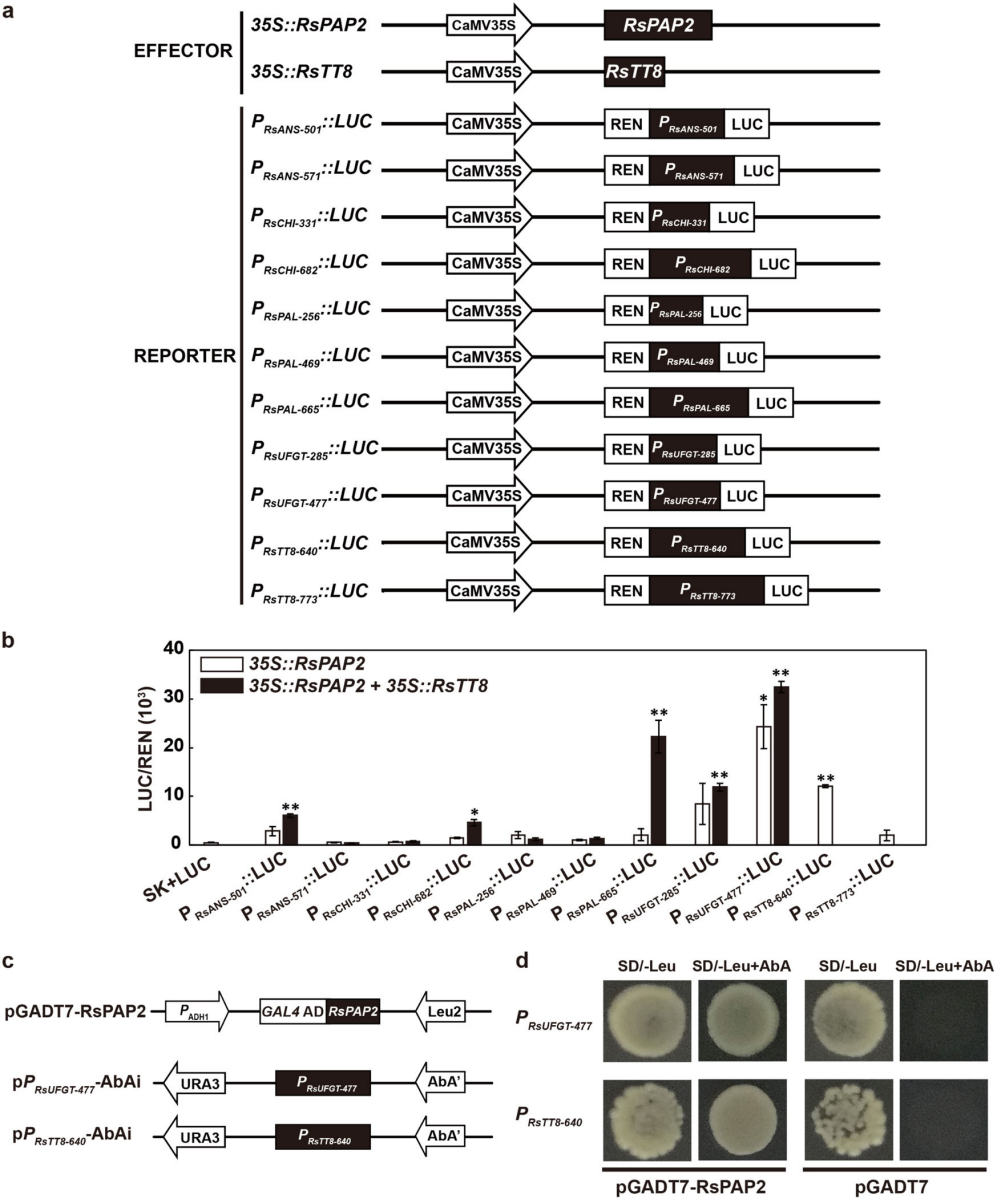
In vivo and vitro interactions of TFs and ATC-related structural gene promoters. **a** Construct details for dual-luciferase assays. **b** The in vivo associations of TFs (*RsPAP2* and *RsTT8*) and promoters were obtained from dual-luciferase assays in tobacco leaves. The REN and LUC activities of different combinations of effector (*35S* *::RsPAP2* and *35S::RsTT8*) and reporter (*P*_*RsANS-501*_*::LUC*, *P*_*RsANS-571*_*::LUC*, *P*_*RsCHI-331*_*::LUC*, *P*_*RsCHI-331/682*_*::LUC*, *P*_*RsPAL-256*_*::LUC*, *P*_*RsPAL-469*_*::LUC*, *P*_*RsPAL-665*_*::LUC*, *P*_*RsUFGT-285*_*::LUC*, *P*_*RsUFGT-477*_*::LUC*, *P*_*RsTT8-640*_*::LUC* and *P*_*RsTT8-773*_*::LUC*) constructs were measured. **c** Construct details for the Y1H assay. **d** Y1H analysis using pGADT7-RsPAP2 as the prey, p*P*_*RsUFGT-477*_-AbAi and p*P*_*RsTT8-640*_*-*AbAi as the bait, and empty pGADT7 with the corresponding recombinant pABAi as a negative control. Error bars represent the SD from three biological replicates in three parallel experiments. Statistical significance was determined using Student’s *t* test. *p < 0.05; **p < 0.01

To examine the ability of RsPAP2 to transactivate the promoters of ATC biosynthetic genes, transient dual-luciferase assays in tobacco leaves were carried out using the constructs shown in Fig. 5a. When *RsPAP2* was transfected alone, the activities of two segmented promoters of the *RsUFGT* (*P*_*RsUFGT-477*_) and *RsTT8* (*P*_*RsTT8-640*_) genes were significantly increased by 47.0- and 22.9-fold, respectively (Fig. 5b). In a yeast one-hybrid (Y1H) system (Fig. 5c), RsPAP2 was further confirmed to directly bind the promoters of *RsUFGT* (*P*_*RsUFGT-477*_) and *RsTT8* (*P*_*RsTT8-640*_) (Fig. 5d).

In addition, stronger activation of *P*_*RsANS-501*_, *P*_*RsCHI-682*_, *P*_*RsPAL665*_, *P*_*RsUFGT-285*_, and *P*_*RsUFGT-477*_ was detected when tobacco leaves were simultaneously cotransformed with *RsPAP2* and *RsTT8* (Fig. 5b), indicating that the RsPAP2–RsTT8 complex might exhibit a broader transcriptional regulatory ability for ATC-related genes. These results indicated that RsPAP2 is an important regulator that selectively induces the expression of several ATC biosynthetic genes to promote ATC accumulation in radish.

## Discussion

Radish taproots provide an excellent source of valuable nutrients, including dietary fiber, sugar, vitamins, and phytochemicals^35,36^. ATCs are responsible for the red skin and flesh color of radish taproots and exhibit nutritional and pharmaceutical properties^22,37^. In a previous study, several genes regulating skin or flesh color in radish taproots have been reported. Based on homology, *RsMYB1* and *RsMYB1a* were separately isolated from the red radish cultivars “Bordeaux” and “Hongxin 1”, and their positive regulation of ATC production was confirmed^22,25^. Transposon-induced methylation of the *RsMYB1* promoter was reported to disturb ATC accumulation in red-fleshed radish^27^. The *RsMyb1*/*RsMYB90* gene is a crucial regulator of radish root skin color^23,26^. Liu et al. reported that the *RsMYB1.1* gene was located within the mapping region for the purple skin of radish root^24^. These findings suggested that MYB TFs are the crucial determinants of ATC accumulation in red-skinned radish and that *MYB* genes from different plant materials might control skin color inheritance via different mechanisms. Therefore, the identification of major genes controlling red skin color across diverse radish genotypes is necessary, which will contribute to revealing the genetic regulation of skin color in radish fleshy roots. The GWAS method plays important roles in the identification of candidate genes related to ATC content in some models and important crop species^14,30,33^. In this study, a GWAS strategy was first applied based on whole-genome sequencing to analyze factors influencing the red skin color of radish.

### GWAS analysis of genetic factors underlying the red skin color of radish

In the present study, a natural radish population containing abundant phenotypic and genetic variations was subjected to GWAS analysis. The GWAS results indicated that there were two leading SNPs on Chr1 and Chr2 associated with red skin color, indicating that at least these two genes might control radish red skin color. Considering LD levels, the candidate gene regions became the focus of efforts to identify key genes related to red skin color^38^. Remarkably, the *RsPAP2* (Rs095840) gene, encoding the R2R3-MYB90 protein, was identified and located on Chr2. Phylogenetic analysis showed that RsPAP2 differed from other published MYBs responsible for skin color in radish and presented a closer relationship with AtMYB90/PAP2 in *Arabidopsis* (Fig. S3a). These results suggested that *RsPAP2* was a novel potential R2R3-MYB gene responsible for red skin color in radish taproots.

### RsPAP2 TF promotes ATC biosynthesis in radish

The *RsPAP2* gene was found to exhibit high transcript levels in all ATC-rich tissues of radish and showed a significant positive correlation with ATC content. To understand the mechanism leading to differential gene expression in red- and white-skinned genotypes, the coding and promoter sequences of the *RsPAP2* gene were compared among the different radish genotypes. It was found that there were three nonsynonymous SNPs in the coding region of *RsPAP2*, but two of the SNPs were only identified in three genotypes with different skin colors, and one SNP was not located in a conserved domain, which similar to the situation of the *SlAN2-like* gene in tomato fruit^39^. However, a total of ten SNPs and four base deletions were found in the 948 bp promoter region, among which two of the base deletions (“T” at 849 bp and “A” at 850 bp) were consistently found among the red-skinned genotypes with the exception of one genotype, “NAU-123” (Table S3). Cases of such insertions or deletions are also found in blood orange, apple, and tomato^39–41^. Although further experimental evidence is needed, this deletion in the promoter region might contribute to the differential expression of *RsPAP2* and affect ATC biosynthesis in radish.

### RsPAP2, either alone or with RsTT8, regulates ATC accumulation in radish

To further understand the positive effect of *RsPAP2* on ATC production in radish, *RsPAP2*-overexpressing *Arabidopsis* lines were obtained via the floral-dip method. The stable expression of *RsPAP2* driven by the CaMV35S promoter in *Arabidopsis* led to ATC accumulation in the roots and leaves, similar to a recent report in *Oilseed rape*^42^. In addition, the radish cotyledons showed distinct accumulation of red pigments after infiltration with *RsPAP2* alone. Moreover, the transcript levels of several key structural genes exhibited significant increases in both *RsPAP2*-overexpressing *Arabidopsis* plants and radish cotyledons. This phenomenon has also been noted for genes in other plants, such as *OvPAP2* in oilseed rape^42^, *NtMYB2* in Chinese narcissus^43^, and *AgMYB2* in purple celery^44^. These findings indicated that *RsPAP2* might promote ATC biosynthesis by positively regulating several critical ATC-related genes in radish. Both dual-luciferase and Y1H assays further confirmed that RsPAP2 could specifically bind to the promoters of *RsUFGT* and *RsTT8*, consistent with previous reports in pear^45^ and grape^46^. In addition, *AtUFGT* and *RsUFGT* genes were intensively upregulated by the overexpression of the *RsPAP2* gene in *Arabidopsis* leaves and radish cotyledons, respectively. This result suggested that the *RsUFGT* gene might be the major control point for ATC accumulation in radish^21^.

**Fig. 6:**
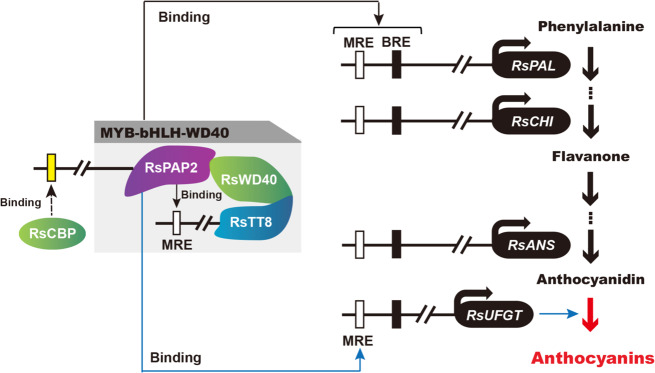
Model for the transcriptional regulation of ATC biosynthesis in radish. The white, black, and yellow boxes represent the MYB-recognizing element (MRE), bHLH-recognizing element (BRE), and CBP binding motif, respectively

In addition, the cotransformation of *RsPAP2* and *RsTT8* demonstrated that the complex could activate the promoters of more structural genes, including *RsPAL*, *RsANS*, *RsCHI*, and *RsUFGT*, indicating that RsTT8 was a partner of RsPAP2 in the regulation of ATC biosynthesis, which might serve as a transcriptional regulatory mechanism of ATC biosynthesis in radish (Fig. 6). RsPAP2 could promote ATC accumulation by binding the MRE element in the *RsUFGT* promoter. It could also act in combination with its cofactor RsTT8 to regulate the activity of additional structural genes (e.g., *RsPAL*, *RsANS*, *RsCHI*, and *RsUFGT*) to promote the ATC biosynthesis pathway. Moreover, it was found that RsPAP2 could function in the transcriptional activation of *RsTT8*, which might be another way to positively increase ATC accumulation in radish. In addition, one SNP within a *calmodulin-binding transcription activator protein* (CBP) gene was detected on Chr2; this SNP was associated with red skin color, and a homolog of this gene, CBP60g, has been reported to regulate the expression of *MYB*s to control ATC biosynthesis in *Arabidopsis*^47,48^. A CBP binding motif was also predicted in the *RsPAP2* promoter region. Therefore, it is possible that RsPAP2, activated by RsCBP, plays an essential role in radish ATC accumulation, which needs to be confirmed by further research.

A previous study indicated that the genetic mechanisms determining radish root skin color differ among different genotypes^24^. Although several ATC-related *RsMYB* genes have been identified by QTL-seq, traditional linkage analysis and genome resequencing using hybrid populations^23,24,26^, the available information is far from complete to fully explain the red skin color variations among diverse radish genotypes. In the present study, to enrich the understanding of ATC biosynthesis regulation, the GWAS approach was first applied to address potential genes responsible for red skin color among various red-skinned radish genotypes. An R2R3-MYB TF, *RsPAP2*, was identified as a candidate gene for red skin color. Lim et al. confirmed that *RsMYB1* is a positive regulator of ATC accumulation in a study involving transgenic *Arabidopsis* and tobacco plants^22^. In our study, based on the observation of transgenic *Arabidopsis* and radish cotyledons, a hypothetical regulatory network of radish ATC biosynthesis regulated by RsPAP2 was proposed by combining the results of dual-luciferase reporter and Y1H assays.

Two R2R3–MYB genes, *RsPAP2* in this study and *RsMYB90* reported previously^26^, were isolated from the same red-skinned radish genotype, “NAU-067” (NAU-YH). In recent years, R2R3-MYB TFs have been proven to be important participants that regulate ATC biosynthesis in several horticultural plants. The SlAN2-like protein combined with SlMYBATV fine-tunes the accumulation of ATCs in tomato fruit. Both *MdMYB10* and *MdMYB110* are regulators of ATC biosynthesis in apple. In this study, the *RsPAP2* gene exhibited high expression levels in tissues (e.g., root skin, sepals, petals, and anthers) with abundant ATC accumulation. Similarly, the expression level of the *RsMYB90* gene was higher in the taproot skin of “NAU-YH” (red-skinned) than that of “NAU-LB” (white skinned). These results indicated that both the *RsPAP2* and *RsMYB90* genes might be crucial regulatory genes involved in ATC biosynthesis in radish. In this study, RsPAP2 was confirmed to directly regulate the transcript levels of the *RsUFGT* gene, and the interaction of RsPAP2 and RsTT8 activated the structural genes *RsPAL*, *RsANS*, *RsCHI*, and *RsUFGT* to promote anthocyanin biosynthesis. Based on the functional analysis of homologous *Arabidopsis* genes, it could be inferred that RsMYB90 might regulate anthocyanin-related genes, including the *PAL* gene of the phenylpropane metabolism pathway. Further functional investigation is required to elucidate the precise roles of the *RsPAP2* and *RsMYB90* genes involved in the regulatory network of skin color formation in radish. This finding of the present study facilitates a deeper understanding of the genetic regulation of red skin color in radish, providing a valuable resource for the genetic improvement of nutritional quality traits in root vegetable crops.

## Materials and methods

### Plant materials and resequencing

A total of 179 cultivated radish genotypes were used in this study. The taproots of these genotypes exhibit a variety of shapes (e.g., circular, elliptic, transverse elliptic, rectangular, narrow rectangular, and pear-shaped) and colors (e.g., white, red, green, purple, and black) (Table S1). Each genotype was planted at the Jiangpu Breeding Station of Nanjing Agricultural University, Nanjing, China. Genomic DNA was extracted from the young leaves of the 179 radish genotypes via the modified CTAB method^30^. Paired-end DNA sequencing libraries with insert sizes ranging from 300 to 500 bp were prepared using the Illumina TruSeq Nano DNA Sample Preparation Kit and sequenced on the Illumina HiSeq 2500 platform.

### SNP calling and population genetic analysis

Raw reads were filtered to remove residual adapters and low-quality bases using SAMtools^49^. The filtered FASTQ files were aligned to the radish reference genome database (WK10039, http://radish-genome.org/)^34^ with Burrows-Wheeler Aligner software^30^. After alignment, the “rmdup” command in the SAMtools package was used to remove PCR duplicates according to the mapping coordinates. The SNPs were filtered with a coverage depth ≥3, a minor allele frequency ≥0.05, and a missing rate <0.2 to obtain high-quality SNPs.

MEGA 6.0 software was used to generate a neighbor-joining phylogenetic tree with 1000 bootstrap replicates^50^. The STRUCTURE program was employed to infer population structure with an admixture model^29^. The simulations were run with a burn-in of 100,000 iterations and a run length of 10^6^ iterations from *K* = 2–7. GCTA software (http://cnsgenomics.com/software/gcta/) was employed to perform PCA of individual genotypes. Haploview software was used to assess the LD coefficient (*r*^2^) between pairwise SNPs across the radish genome^51^.

### GWAS analysis and candidate gene identification

The association analysis was performed with the Genome-wide Efficient Mixed-Model Association program based on the SNP data obtained through resequencing^52^. The significance threshold was estimated as −log_10_(*P*) = 7.0 to identify significantly associated SNPs, which was between the Bonferroni correction value of this study (−log_10_(*P*) = 7.4, *α* = 0.05) and the threshold (−log_10_(*P*) = 6.0, *α* = 0.05) applied in other similar studies^14,29^.

The physical positions of significantly associated SNPs (−log_10_ *P* > 7.0) for target traits were used to identify potential candidate genes in the radish genome^34^. The interval for candidate gene identification was ±10 kb around the SNPs associated with the target traits. The Haploview package was employed to estimate the candidate regions by pairwise LD correlation^53^.

### Gene expression analysis

A total of nine genotypes with different root sizes, shapes, and colors were selected for RNA extraction, which included “NAU-026”, “NAU-062”, “NAU-067”, “NAU-069”, “NAU-074”, “NAU-112”, “NAU-118”, “NAU-136”, and “NAU-152”. Using an RNAprep Pure Plant Kit (Tiangen, China), total RNA was isolated from different tissues of the following two groups: the first comprised the root skin and flesh of nine genotypes, and the second comprised the root skin, leaf vein, stem, sepal, petal and anther tissues of two genotypes with red skin and white skin (“NAU-067” and “NAU-069”). The results for the two groups were employed to examine the expression pattern of the candidate *RsPAP2* gene.

For RT-qPCR analysis, first-strand cDNA was synthesized from total RNA using the PrimeScript™ RT reagent kit with gDNA Eraser (Takara, Dalian, China). Gene expression signatures were generated from three biological replicates in three parallel experiments employing an RT-qPCR approach in a LightCycler^®^ 480 System (Roche, Mannheim, Germany) according to the manufacturer’s instructions. The relative expression levels were normalized to those of the *Actin* gene for radish and the elongation factor 1 alpha (*EF1α*) gene for *Arabidopsis* as internal references and calculated by the $2^{-\Delta \Delta {\rm{C}}_{\rm{T}}}$ method^54^. All primers used for RT-qPCR analysis are listed in Table S4.

### Sequence analysis

The protein sequences of the published radish MYBs were downloaded from three previously published radish genome datasets^34,35,55^ and the NCBI database (https://www.ncbi.nlm.nih.gov/). Related protein sequences of *Arabidopsis* were obtained from the TAIR database (https://www.arabidopsis.org/). Clustal X and MEGA 6.0 software were used for multiple sequence alignment and phylogenetic analysis, respectively^49,56^.

Conserved *cis*-acting regulatory elements located in promoter regions were scanned by using PlantCARE (http://bioinformatics.psb.ugent.be/webtools/plantcare/html/), combined with previous reports^11^.

### Overexpression vector construction and *Arabidopsis* transformation

The full-length coding sequence (CDS) of *RsPAP2* was isolated from the red radish genotype “NAU-067” and cloned into the pCAMBIA-2301 vector containing the CaMV35S promoter and the GUS reporter gene using the traditional digestion and ligation method with restriction endonucleases (*Bam*H I and *Kpn* I) and T4 ligase (Takara, Dalian, China). The recombinant vector was introduced into *Agrobacterium tumefaciens* strain GV3101 (WeiDi, Shanghai, China). The genetic transformation of *Arabidopsis* was conducted using the floral-dip method^42^. Transgenic *Arabidopsis* plants carrying the *RsPAP2* gene were identified by selection on half-strength Murashige and Skoog agar plates containing 100 mg/L kanamycin, along with assays for β-glucuronidase activity and RT-qPCR amplification. All primers used for vector construction are shown in Table S4. The leaves and roots of the transgenic *Arabidopsis* lines were sampled for the measurement of total ATC contents and RT-qPCR analysis.

### Induction of anthocyanins in transiently transformed radish cotyledons

The full coding region of *RsPAP2* was cloned into the multiple cloning sites of the pGreenII 62-SK vectors. The recombinant plasmid was transformed into *Agrobacterium* strain GV3101 (pSoup) using the freeze–thaw method. The *Agrobacterium* strain containing *RsPAP2* was infiltrated into the abaxial cotyledon surface of the red-skinned radish genotype “NAU-067”^57^. Cotyledons transformed with pGreenII 62-SK containing a noncoding sequence were used as the control. The phenotype of the cotyledons was monitored and photographed at 9 d postinfiltration. The infiltrated cotyledon samples were collected for the determination of total ATC contents and RT-qPCR analysis. The primers used for vector construction are shown in Table S4.

### Anthocyanin analysis

A reported methanol-HCl method with minor modification was used to extract total ATC^9^. Approximately 0.1 g of a sample was soaked and incubated for 12 h in a tube containing 5 mL of a methanol and 0.1% (v/v) HCl solution in the dark at room temperature. The ATC concentration was measured and calculated as described by Li et al.^58^. All samples were measured in triplicate with three independent biological replicates.

### Dual-luciferase assay of transiently transformed tobacco leaves

The full-length CDS of *RsTT8* was isolated from the red radish genotype “NAU-067” and cloned into the multiple cloning sites of the pGreenII 62-SK vectors. Segmented promoters of ATC biosynthetic genes containing putative MRE and BRE elements were inserted into the cloning site of pGreenII 0800-LUC. All constructs were transformed into *Agrobacterium* strain GV3101 (pSoup). *Agrobacterium* culture mixtures of TFs (*RsPAP2*, *RsPAP2* and *RsTT8*) and promoters (10:1) were infiltrated into 4-week-old tobacco (*Nicotiana benthamiana*) leaves by using needleless syringes. The infiltration, transient expression analysis, and enzyme activity quantification of Firefly luciferase (LUC) and Renilla luciferase (REN) were conducted. Three days after infiltration, LUC and REN activities were analyzed using a Dual-Luciferase Reporter Assay System (Promega, Wisconsin, USA). Finally, TF-promoter interactions were measured as the ratio of LUC to REN in three independent experiments with at least three biological replicates for each assay. The primers used for the dual-luciferase assay are shown in Table S4.

### Y1H assay

Y1H assays were performed using the Matchmaker Gold Yeast One-Hybrid System Kit (Clontech, Palo Alto, USA) to evaluate the binding of RsPAP2 to the MRE and BRE elements in ATC-related gene promoters. These amplified promoter sequences were cloned into the pAbAi vector to generate the bait constructs. The coding region of *RsPAP2* was fused in frame with the GAL4 activation domain (AD) in the pGADT7 AD vector to generate the prey vector (pGADT7-RsPAP2). Bait vectors linearized by *Bst*B I were transformed into the Y1HGold yeast strain to screen the minimal inhibitory concentrations of aureobasidin A (AbA) (Solarbio, Beijing, China). Then, pGADT7-RsPAP2 was introduced into the Y1HGold yeast strain on media lacking Leu (SD/−Leu) supplemented with optimal AbA (100 and 350 ng/mL). Empty pGADT7 with the corresponding recombinant pABAi was used as a negative control. The primers used for the Y1H assay are shown in Table S4.

## Supplementary information

Supplemental Figures

Table S1

Table S2

Table S3

Table S4

## Data Availability

All raw sequences of the 179 radish genotypes have been deposited in the Sequence Read Archive of the National Center for Biotechnology Information under BioProject number PRJNA634208. The coding sequence of the *RsPAP2* gene from NAU-067 has been deposited in GenBank under accession number MT459822.
