# Supplementary material for: A genome-wide association study uncovers a critical role of the RsPAP2 gene in red-skinned Raphanus sativus L
Source: Hortic Res. 2020 Sep 24;7:164. doi: 10.1038/s41438-020-00385-y (PMC7518265; doi:10.1038/s41438-020-00385-y)
Supplement: Supplementary file 1 — Supplemental Figures [file 41438_2020_385_MOESM1_ESM.docx]

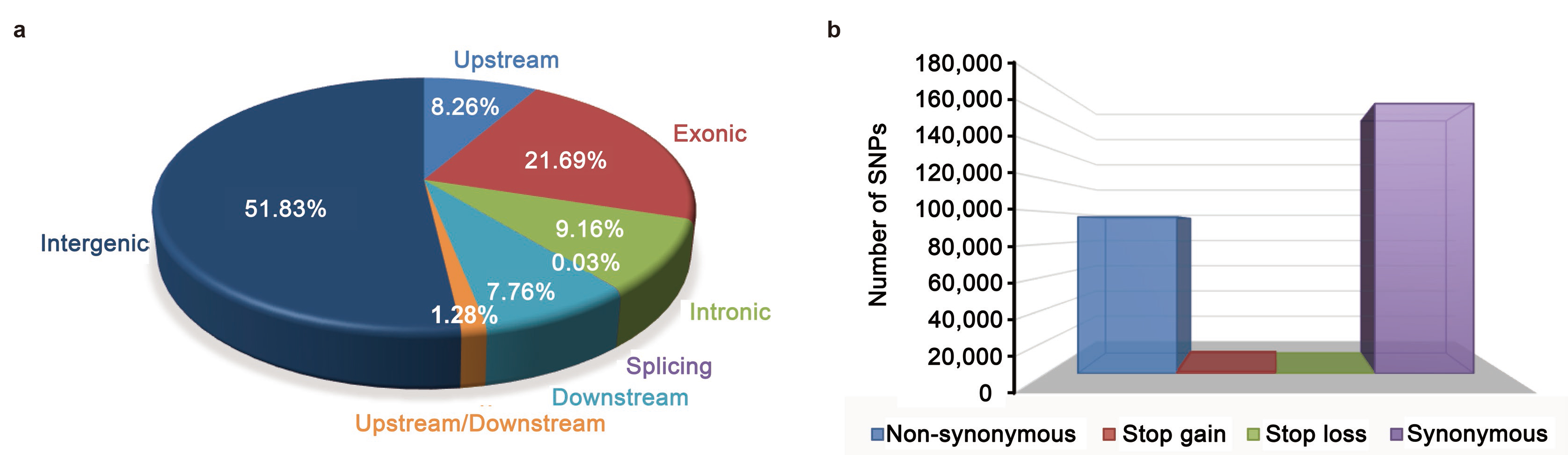


**Fig. S1 SNP detection and annotations statistics in 179 radish genotypes by resequencing.**


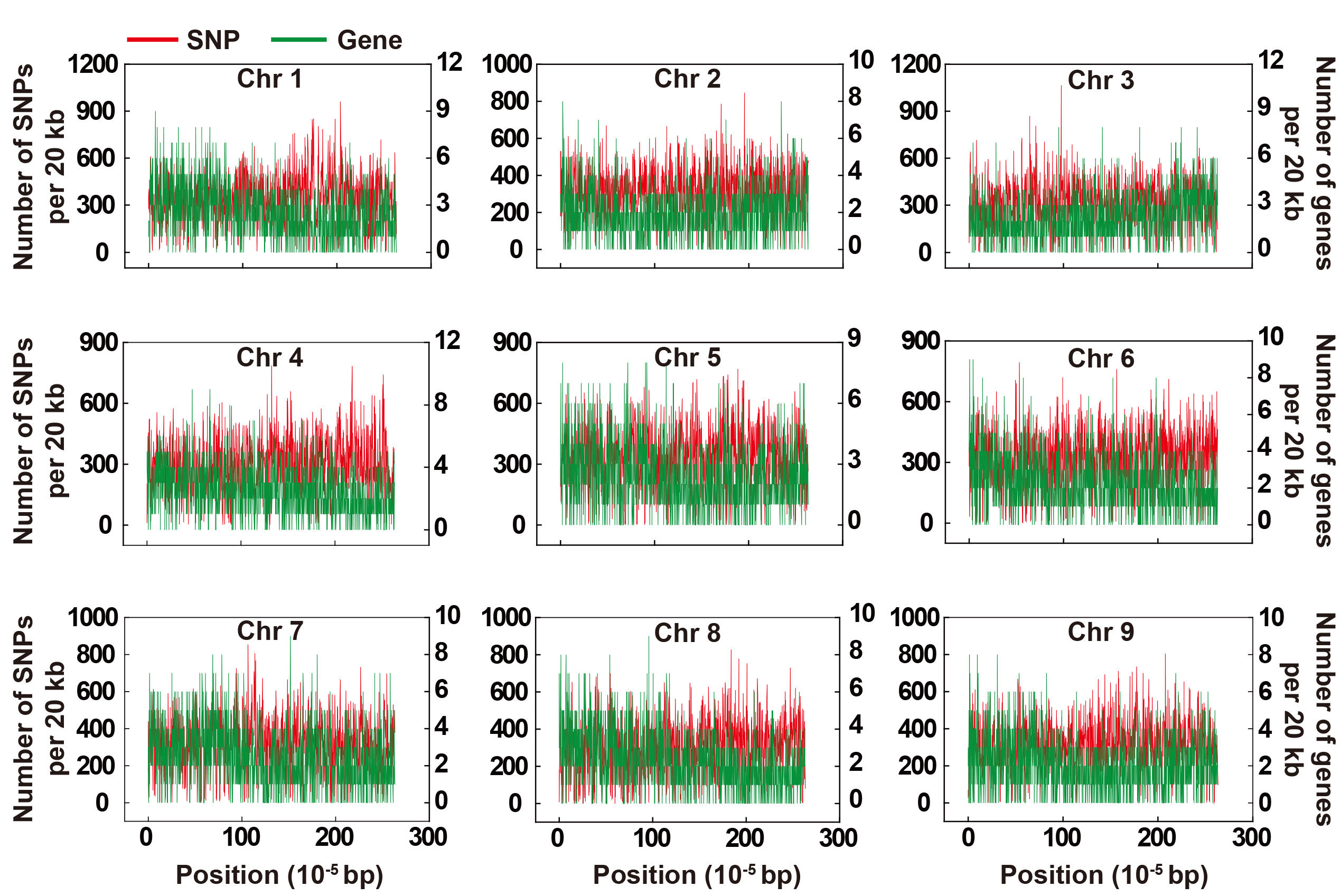


**Fig. S2 Distribution of SNPs and genes across the nine chromosomes in radish.**


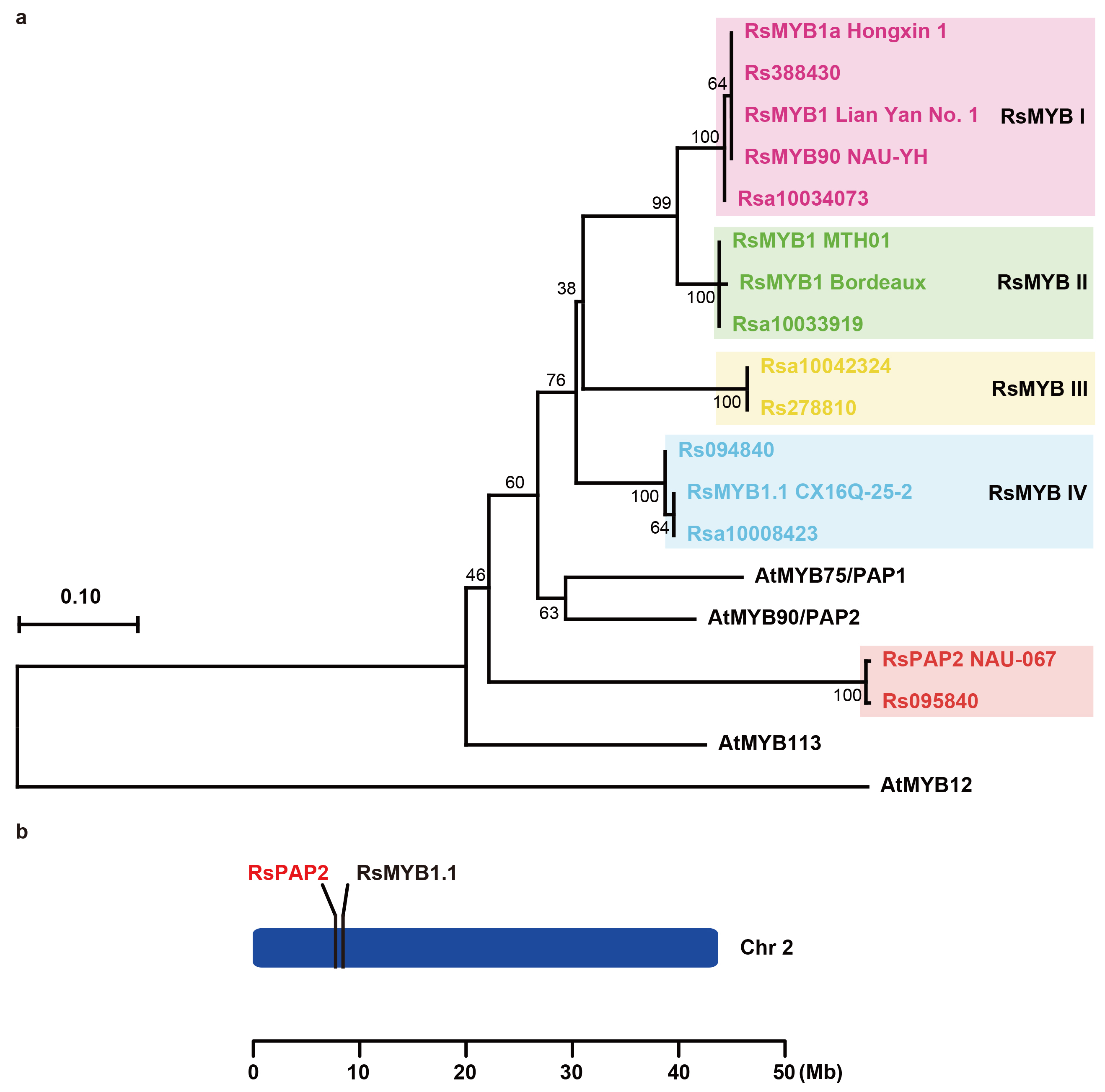


**Fig. S3 Protein sequence analysis of *RsMYB* genes in radish and ATC-related *R2R3-MYB*s in *Arabidopsis*.** (a) Phylogenetic tree of these MYBs. (b) The chromosome locations of RsPAP2 and RsMYB1.1 in the “WK10039” radish genomes. The accession number of these proteins are as follows: AtMYB113, AT1G66370; AtMYB12, AT2G47460; AtMYB75/PAP1, AT1G56650; AtMYB90/PAP2, AT1G66390; RsMYB90 NAU-YH and RsMYB1 Lian Yan No. 1, Rs388430; RsMYB1.1 CX16Q-25-2, Rsa10008423; RsMYB1 Bordeaux, KR706195; RsMYB1 MTH01, MN308185; RsMYB1a Hongxin 1, RSG19108.t1; RsPAP2 NAU-067, MT459822.


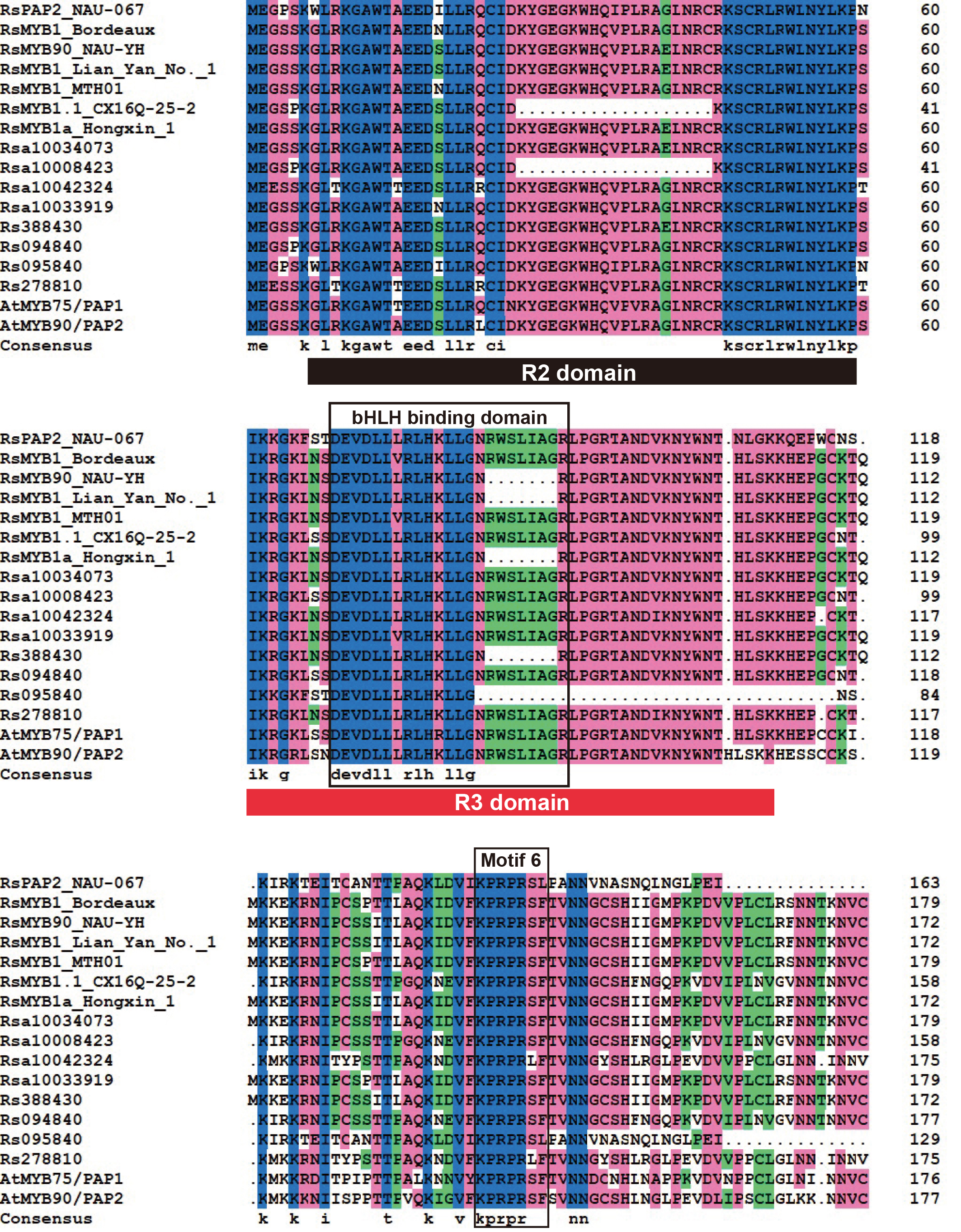


**Fig. S4 Protein sequence alignment of *RsPAP2* and the other known ATC-related *MYB*s from radish and *Arabidopsis*.** The R2 and R3 domains are underlined by black and red, respectively. The bHLH-binding motif is boxed in the R3 domain. Motif 6 was previously identified in the C-terminal domain of ATC-related MYBs.


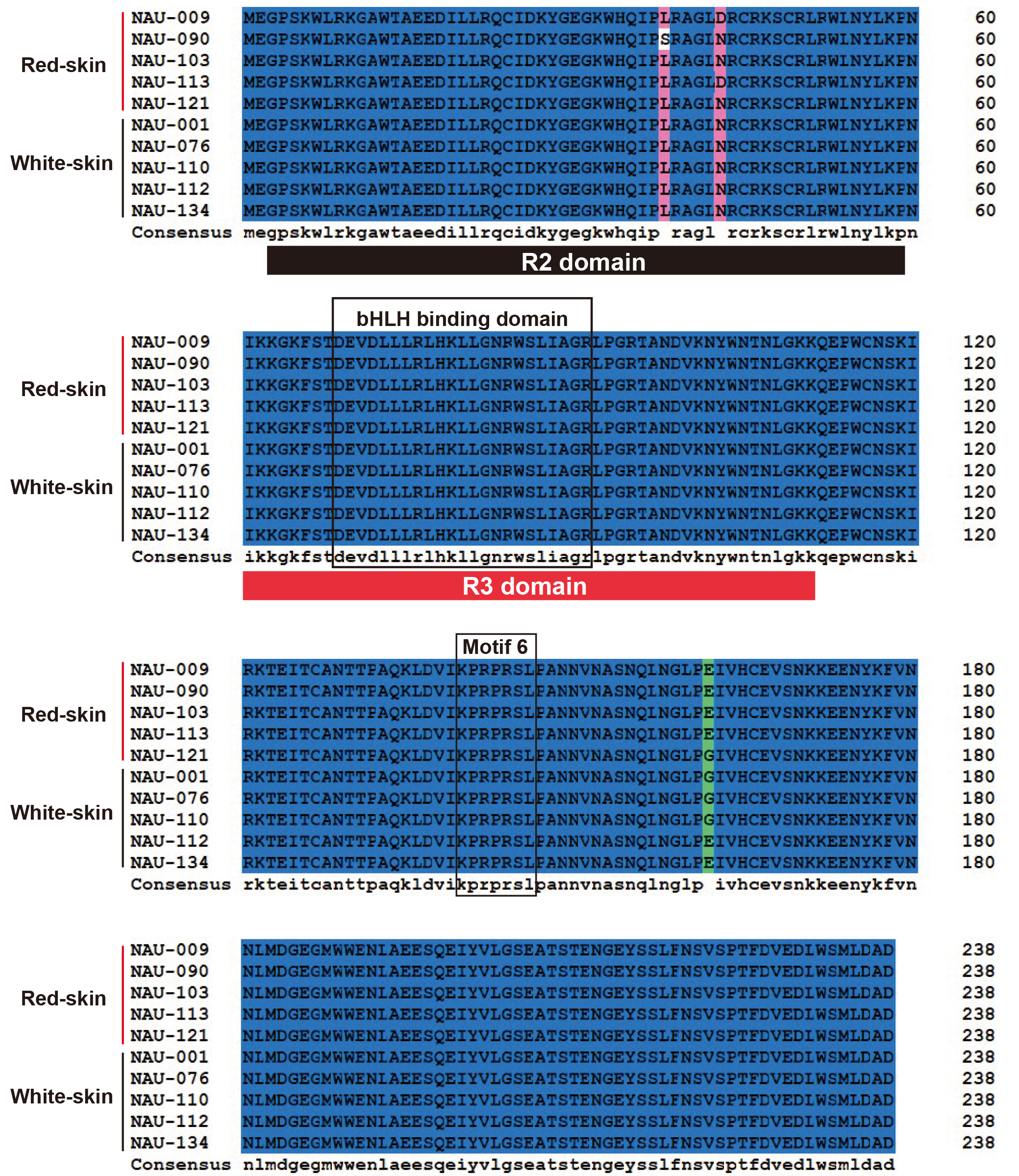


**Fig. S5 Amino acid sequences of *RsPAP2* from five red-skin and five green-skin radish genotypes.** The R2 and R3 domains are underlined by black and red, respectively. The bHLH-binding motif is boxed in the R3 domain. Motif 6 was previously identified in the C-terminal domain of ATC-related MYBs.


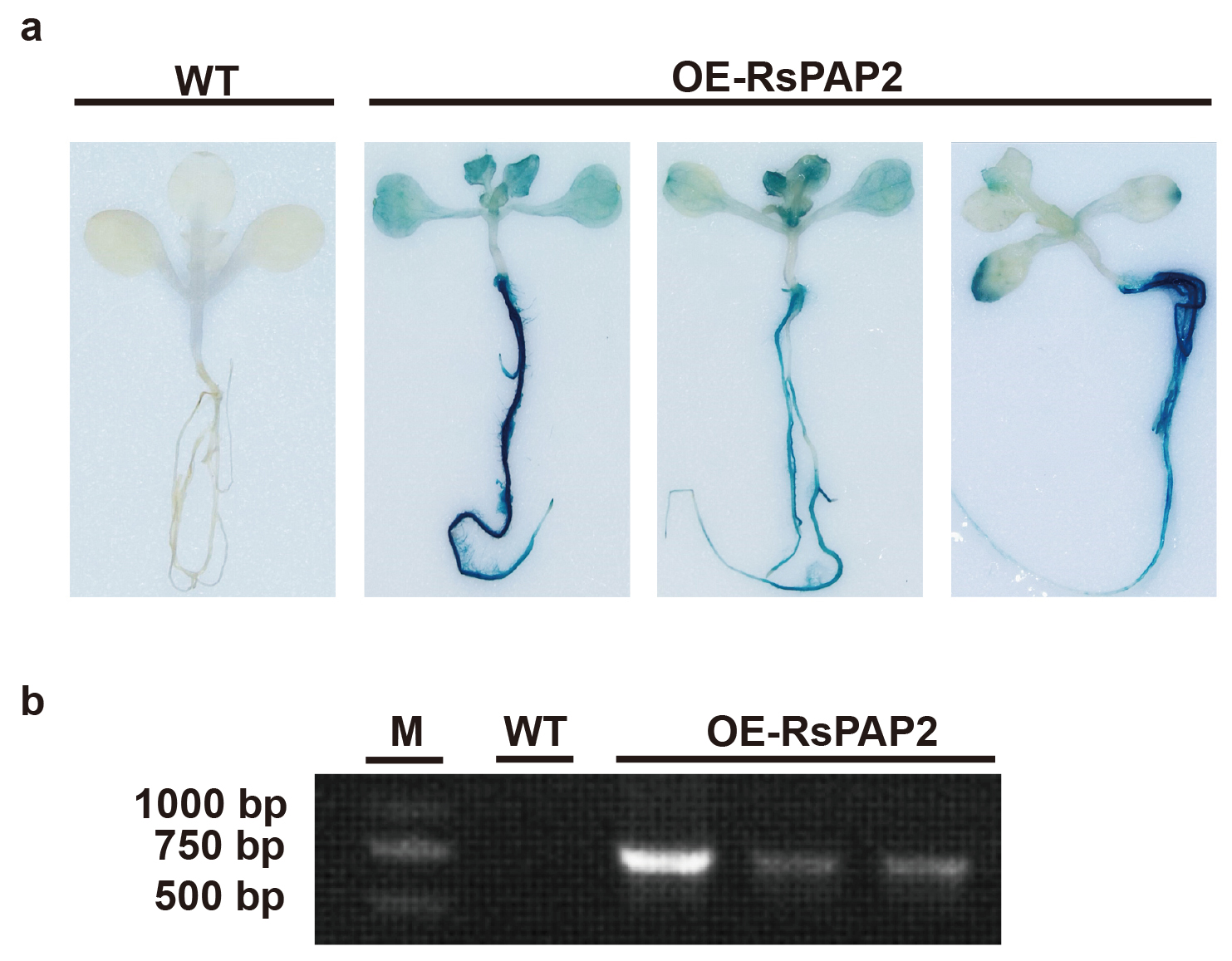


**Fig. S6 Histochemical GUS (a) and PCR (b) assays of WT and transgenic *Arabidopsis* seedlings.** M, Marker; WT, Wild type; OE-RsPAP2, RsPAP2-overexpressing *Arabidopsis* plant.
