# Supplementary material for: A genome-wide association study uncovers a critical role of the RsPAP2 gene in red-skinned Raphanus sativus L
Source: Hortic Res. 2020 Sep 24;7:164. doi: 10.1038/s41438-020-00385-y (PMC7518265; doi:10.1038/s41438-020-00385-y)
Supplement: Supplementary file 3 — Table S2 [file 41438_2020_385_MOESM3_ESM.docx]

**Table S2** Summary of peak SNPs and candidate genes for GWAS analysis on red-skin color of radish.

| **Signal**  **index** | **Chr.** | **Start** | **End** | **SNP number** | **Peak Position** | **Reference/ Alternative** | **MAF** | **-Log_10_ *P*** | **Candidate genes** | **Annotation** |
| --- | --- | --- | --- | --- | --- | --- | --- | --- | --- | --- |
| 1 | 1 | 20960187 | 20980187 | 1 | 20970187 | C/G | 0.49 | 8.04 | Rs033010  Rs033020 | DNA binding  EamA-like transporter family protein |
| 2 | 1 | 20974537 | 20994537 | 1 | 20984537 | C/G | 0.49 | 7.88 | Rs033050  Rs033060  Rs033080  Rs033090 | CBL-interacting protein kinase 17  Unknown protein  Unknown protein  Unknown protein |
| 3 | 1 | 20992097 | 21022646 | 6 | 21012529 | G/T | 0.49 | 9.42 | Rs033100  Rs033110  Rs033130  Rs033140 | Ribonuclease H-like superfamily protein  Unknown protein  Unknown protein  Hypothetical protein CARUB_v10026382mg |
| 4 | 1 | 21041213 | 21062436 | 3 | 21052436 | C/A | 0.49 | 8.69 | - | - |
| 5 | 1 | 21054249 | 21076334 | 3 | 21064249 | T/C | 0.46 | 8.87 | Rs033160  Rs033170  Rs033180  Rs033190 | Unknown protein  G-protein-coupled receptor 1  Unknown protein  Hydroxyproline-rich glycoprotein family protein |
| 6 | 2 | 7655845 | 7675845 | 1 | 7665845 | T/C | 0.40 | 7.40 | Rs095910  Rs095920  Rs095930  Rs095940 | Uncharacterized protein  Ribonuclease H-like superfamily protein  Unknown protein  HCP-like superfamily protein |
| 7 | 2 | 7717557 | 7737557 | 1 | 7727557 | G/T | 0.35 | 7.83 | Rs095840 | Myb domain protein 90 |
| 8 | 2 | 7755197 | 7775197 | 1 | 7765197 | T/A | 0.14 | 7.92 | Rs095770  Rs095780  Rs095790  Rs095800 | Glyoxal oxidase-related protein  Calmodulin-binding transcription activator protein  DNA primase, large subunit family  Unknown protein |
| 9 | 3 | 26962457 | 26982458 | 2 | 26972458 | C/A | 0.13 | 7.25 | Rs144230  Rs144260  Rs144280 | MATE efflux family protein  Zinc finger protein-related  Myb domain protein 92 |
| 10 | 6 | 14513023 | 14533043 | 2 | 14523043 | G/A | 0.33 | 7.39 | Rs312800 | alpha/beta-Hydrolases superfamily protein |
| 11 | 6 | 21763152 | 21783152 | 1 | 21773152 | G/C | 0.10 | 8.50 | Rs336820  Rs336830 | Syntaxin/t-SNARE family protein  Molecular function unknown |
| 12 | 7 | 9290017 | 9310017 | 1 | 9300017 | T/C | 0.45 | 7.11 | Rs388520  Rs388530  Rs388540 | Unknown protein  Duplicated homeodomain-like superfamily protein  Duplicated homeodomain-like superfamily protein |
| 13 | 9 | 3864762 | 3884762 | 1 | 3874762 | A/G | 0.35 | 7.29 | Rs450930  Rs454150 | Auxin efflux carrier family protein  TPX2 (targeting protein for Xklp2) protein family |
| 14 | 9 | 23521554 | 23541556 | 2 | 23531554 | C/T | 0.20 | 8.44 | Rs480530  Rs480540  Rs480550  Rs480560  Rs480570 | Thylakoid rhodanese-like  Ubiquitin carboxyl-terminal hydrolase family protein  pentatricopeptide (PPR) repeat-containing protein  PYR1-like 7  Ubiquitin-like superfamily protein |
