# Supplementary material for: A genome-wide association study uncovers a critical role of the RsPAP2 gene in red-skinned Raphanus sativus L
Source: Hortic Res. 2020 Sep 24;7:164. doi: 10.1038/s41438-020-00385-y (PMC7518265; doi:10.1038/s41438-020-00385-y)
Supplement: Supplementary file 4 — Table S3 [file 41438_2020_385_MOESM4_ESM.docx]

**Table S3** The SNPs and deletions of the promoter sequences of *RsPAP2* in different radish genotypes.

| **Color of radishes** | **Individual code** | **Locations of distance transcription start site of *RsPAP2* (bp)** | | | | | | | | | | | | | |
| --- | --- | --- | --- | --- | --- | --- | --- | --- | --- | --- | --- | --- | --- | --- | --- |
|  |  | **- 27** | **- 61** | **- 180** | **-186** | **-245** | **-333** | **-354** | **- 460** | **- 585** | **- 598** | **- 603** | **- 679** | **- 849** | **- 850** |
| Red skin | NAU-009 | . | A | A | A | A | T | T | A | A | T | A | T | . | . |
|  | NAU-090 | A | A | A | A | A | T | T | A | G | T | A | T | . | . |
|  | NAU-103 | . | A | A | A | A | T | T | A | A | T | A | . | . | . |
|  | NAU-113 | A | A | A | A | A | T | T | A | A | T | A | T | . | . |
|  | NAU-121 | . | A | A | G | G | T | C | A | A | T | A | T | . | . |
|  | NAU-123 | A | A | A | A | A | T | T | G | A | T | A | T | T | A |
|  | NAU-130 | A | A | G | A | A | T | T | A | A | T | A | . | . | . |
|  | NAU-131 | A | G | A | A | A | T | T | A | A | T | G | . | . | . |
|  | NAU-132 | . | A | A | A | A | T | T | A | A | T | A | T | . | . |
|  | NAU-152 | . | A | A | A | A | T | T | A | A | T | A | T | . | . |
| White skin | NAU-076 | A | A | A | A | A | T | T | A | A | T | A | T | T | A |
|  | NAU-110 | A | A | A | A | A | T | T | A | A | T | A | T | T | A |
|  | NAU-112 | A | A | A | A | A | T | T | A | A | T | A | . | T | A |
|  | NAU-134 | . | A | A | A | A | T | T | A | A | T | A | T | . | . |
|  | NAU-135 | A | A | A | A | A | T | T | A | A | T | A | T | . | . |
|  | NAU-143 | . | A | A | A | A | T | T | A | A | T | A | T | T | A |
|  | NAU-144 | . | A | A | A | A | C | T | A | A | C | A | T | T | A |
|  | WK10039 | A | A | A | A | A | T | T | A | A | T | A | T | T | A |
