# Supplementary material for: A genome-wide association study uncovers a critical role of the RsPAP2 gene in red-skinned Raphanus sativus L
Source: Hortic Res. 2020 Sep 24;7:164. doi: 10.1038/s41438-020-00385-y (PMC7518265; doi:10.1038/s41438-020-00385-y)
Supplement: Supplementary file 5 — Table S4 [file 41438_2020_385_MOESM5_ESM.docx]

**Table S4** List of primer sequences in this study.

| **Gene name** | **Forward primer (5’- 3’)** | **Reverse primer (5’- 3’)** | **Application** |
| --- | --- | --- | --- |
| *At4CL* | TGGTGCTGCTCCTCTTGG | CTGGACCTGCTTCCGTCA | RT-qPCR |
| *AtANS* | GGGTTGTGAAAGATGCTAAATTA | AGTTTTCTCACTTCTTCTCCGTAC | RT-qPCR |
| *AtC4H* | CTGAGCAGAAGGGAGAAATCA | ATTCCCCACTCGATAGACCA | RT-qPCR |
| *AtCHI* | ATCTATCCCGTTCTTCCGTG | TATAAAGCCCTAATTGTTTCCATA | RT-qPCR |
| *AtCHS* | TGGCACTGCTAACCCTGAGA | GCATGTGACGTTTCCGAATT | RT-qPCR |
| *AtDFR* | GTTGCTTCTTGGCTCATCATG | ATGCAAATGGTAGCTCGGTTA | RT-qPCR |
| *AtF3H* | TGACTCGTCTCGCTCGTGA | TCTCCAATCTTGCACAGCCT | RT-qPCR |
| *AtMYB113* | TAAGTATGGAGAAGGCAAATGG | AAGTTTATGAAGGCGAAGAACA | RT-qPCR |
| *AtPAL1* | TGGTGCTACTTCTCATCGGAG | GGCTTGTTTCTTTCGTGCTT | RT-qPCR |
| *AtPAP2* | CAAATGGCATCAAGTTCCTTT | TAGAAGCTTATGAAGGCGAAGA | RT-qPCR |
| *AtTT8* | GAGGATTTGACGGAGACAGAA | TGTCAACTTCATTTGCACCAC | RT-qPCR |
| *AtUFGT* | TCCCAAGAAAGCACTAAACCAG | CCAATGGGCAGAATCATAGAAA | RT-qPCR |
| *AtWD40* | GCCAATCTCGGTTCTCAACA | TTTCGGCTCTACATCGTTCC | RT-qPCR |
| *AtEF1a* | GCCACACCTCTCACATTG | TACCAGCGTCACCATTCT | RT-qPCR |
| *Rs4CL* | GCCTAATCAACGGTCCCAC | TTTCAATCCTTCGCTTCCTG | RT-qPCR |
| *RsActin* | GCATCACACTTTCTACAAC | CCTGGATAGCAACATACAT | RT-qPCR |
| *RsANS* | TCCATAAGAAACCCTTCCAAGT | TGACAGTGTCTCCACCAACCT | RT-qPCR |
| *RsC4H* | CGTTCCTTAGAGGCTACTTGAAG | TTTCAATCCTTCGCTTCCTG | RT-qPCR |
| *RsCHI* | TTATCTAGCTCCTGTCGCCGT | CCACACAGTTCTCCGTTACTTTCT | RT-qPCR |
| *RsF3H* | GGCTCGTGACTTCTTCGC | TAGTCGGCCACCGTGAGT | RT-qPCR |
| *RsPAL* | ACGGTGTCGCACTTCAGAAG | CCGGAGTAGCCTTGGAGGA | RT-qPCR |
| *RsPAP2* | GCTGTAGACTAAGATGGCTGAAC | GTAATGATCGAGGTCGAGGTT | RT-qPCR |
| *RsTT8* | TCTGGCAACTTTGTCCTCAAC | CAGCCTCTTCAGCCGTTATTT | RT-qPCR |
| *RsUFGT* | TTTTCTTGACCCATTTGCTTG | TCCAATCAGCGGAATCGTAG | RT-qPCR |
| *RsWD40* | TACCCTCCCACCAAGCTC | GTCTTGCTGTTGTTGAGGACC | RT-qPCR |
| *RsPAP2* | CGGGATCCATGGAAGGTCCATCCAAATGG | GGGGTACCTCATGTATCTGCGTCGAGCATAC | Cloning for CDS |
| *RsTT8* | CGGAATTCATGGATGAATCAAGTATTATACCG | GGGGTACCCTAGAGTTTATTTTGAGATATG | Cloning for CDS |
| *RsANS*-501pro | GGGGTACCGAAAGGTAAATTGTTCACTTTGTG | CGGGATCCGTGGGGGTATAAATATGGAAAA | Dual-luciferase assay |
| *RsANS*-571pro | GGGGTACCTAGGGGTAGGCACTTCGATT | CGGGATCCAATAACTAGGGGTGGGCAAA | Dual-luciferase assay |
| *RsCHI*-331pro | GGGGTACCATTGTGGTCCAAGTTCACATAAG | CGGGATCCGGGTCAAGATCTAGTACCTCTTAAAT | Dual-luciferase assay |
| *RsCHI*-682pro | GGGGTACCCCATTAAATTAGCCAAAAATCTT | CG*GGATCC*TCGTTTCAGCTTAGGTTTAAGA | Dual-luciferase assay |
| *RsPAL*-256pro | GGGGTACCTATCACTAACTTTACTTCCATATATTTACTC | CGGGATCCTTTTAGTATAATAATTTGCCTTGTCA | Dual-luciferase assay |
| *RsPAL*-469pro | GGGGTACCAATTGCTTTTGTCATGCAAAC | CGGGATCCTTTCATAAGATATTTTTTTGATTTTG | Dual-luciferase assay |
| *RsPAL*-665pro | GGGGTACCTACCACCACAATGAAACGCT | CGGGATCCTTGGTCCTGAGGGAAAAGAG | Dual-luciferase assay |
| RsTT8-640pro | GGGGTACCCTCACATACCACATACCCAACTG | CGGGATCCGCTTTTATGCGTTCTGACTGG | Dual-luciferase assay |
| RsTT8-773pro | GGGGTACCGGTGGGTAGAGTTATGAAAATGTAG | CGGGATCCTAACGGCCACTTGGATTGA | Dual-luciferase assay |
| *RsUFGT*-285pro | GGGGTACCCAACAATAACTCGTTGAACAAAAA | CGGGATCCCTTATGTGTTTGGGTGTTTCTGA | Dual-luciferase assay |
| *RsUFGT*-477pro | GGGGTACCTTTGGTCTTGACAATGGGAATA | CGGGATCCTTGGTGTTGGCGATGCTG | Dual-luciferase assay |
| *RsPAL*-665pro | CCAAGCTTTACCACCACAATGAAACGCT | GGGGTACCTTGGTCCTGAGGGAAAAGAG | Y1H assay (BD) |
| *RsPAP2* | CCCCCGGGATGGAAGGTCCATCCAAATGG | CGGGATCCTCATGTATCTGCGTCGAGCATAC | Y1H assay (AD) |
| RsTT8-640pro | CCAAGCTTCTCACATACCACATACCCAACTG | GGGGTACCGCTTTTATGCGTTCTGACTGG | Y1H assay (BD) |
| RsUFGT-285pro | CCAAGCTTCAACAATAACTCGTTGAACAAAAA | GGGGTACCCTTATGTGTTTGGGTGTTTCTGA | Y1H assay (BD) |
| RsUFGT-477pro | CCAAGCTTTTTGGTCTTGACAATGGGAATA | GGGGTACCTTGGTGTTGGCGATGCTG | Y1H assay (BD) |
